# Becoming More Open: Increasing Rigor and Reproducibility of Gerontological Science

**DOI:** 10.1093/geroni/igaa057.1877

**Published:** 2020-12-16

**Authors:** Brad Taylor, Lewina Lee, Derek Isaacowitz

## Abstract

“Open science” refers to a collection of practices with the broad goal of increasing the transparency and reproducibility of scholarly research. The purpose of this symposium is to illustrate the application of open science practices to gerontological research and address barriers to adopting such practices. Drawing from his experiences in leading a research network that conducts integrative analysis of longitudinal aging studies (IALSA), Hofer will describe challenges posed by multiple sources of heterogeneity in conducting coordinated analyses, and ways of handling these challenges to maximize reproducibility. Next, Mroczek will illustrate these issues by providing two examples of coordinated analyses. This talk will highlight design features that promote openness and transparency in conducting research on longitudinal data. Third, Lodi-Smith will provide practical guidance and examples on preregistering complex projects, strategies for transparently reporting deviations from preregistrations, considerations in sharing sensitive data, and tips on transparent documentation of analysis code. She will also emphasize the pedagogical value of preregistration. Finally, Seaman will describe ongoing efforts to establish open science practices as the default in her laboratory, with the goal of providing a model for both junior and more established researchers wanting to build transparency into their research practices. Discussant Isaacowitz, editor for the Journal of Gerontology, Series B: Psychological Sciences, will evaluate the presentations from the lens of how journals can encourage more transparent and replicable scientific practices.

